# 

**Published:** 1835-01-01

**Authors:** 


### METEOROLOGICAL REGISTER,

### NOTICES.

MR. VALENTINE *will see that we have taken advantage of one of his suggestions; the other is still under consideration.*

*We regret that we cannot comply with the requests of* DR. COWAN *and* MR. SPENDER; *but literary intelligence appears only in our Advertising Sheet.*

*We are much obliged to* DR. STANLEY *for his paper on Animal Magnetism: he will find a notice of it in our Collectanea.*

*The Review of* GRIFFIN *may be obtained by the author, from our publisher's.*

*We have received the following Books:*

FORBES'S *Translation of* LAENNEC, *fourth Edition.*
DUFFIN *on Lateral Deformity of the Spine.*
CLOQUET *on Inguinal and Femoral Hernia, translated by* MR. M'WHINNIE.
PETTIGREW'S *Clinical Lecture on a Case of Hydrophobia.*
CURTIS'S *Essay on the Deaf and Dumb. Second Edition.*
FORD *on Dropsy.*
MARSDEN *on Cholera.*
GEORGE *on Cholera.*
WAITE *on the Gums.*
MACILWAIN'S *Introductory Lecture.*
LEESE *on Gout.*
GAITSKELL *on Mental Derangement.*

*We shall review several of them in our next.*

### INDEX TO VOL. III.

PAGE

ABERCROMBIE on Diseases of the Brain (third edition), reviewed . 301 *et seq.*
Acupuncture and Galvanism . . . . . 472
Address . . . . . . . 1
Ague, Septan . . . . . . . 209
Alcohol, its effect on the mucous membrane of the stomach . 70
Amaurosis, case of, cured by Strychnine . . . 225
Ammonia, carbonate of, in the urine . . . 470
Amputation in cases of Gunshot Wounds, Dupuytren on . . 134
Ana, or āā ; explanation of this abbreviation . . 157 & 8
Anatomy, Elements of, by Dr. Quain, reviewed . . 369 *et seq.*
for Artists, by George Simpson, M.R.C.S., reviewed . 161
of the Bladder, Mr. Guthrie's account of the . . 3
Aneurism, various methods of treating . . . 213
Aneurisms of the Cerebral Arteries, Mr. Thomas King on . . 434
Animal Magnetism . . . . . 459
Anus, operation for imperforate . . . . 202
Aorta tied by Dr. John Murray . . . . 404
Arachnoidea oculi . . . . . 325
Aretæus knew that the arteries contained blood . . 398
Aristotle quoted . . . . . . 360
Arsenic, antidote to . . . . . . 417
Arteriotomy, best method of . . . . . 278
Artichoke, extract of, used in rheumatism . . . 420
Asphyxia, Dr. Kay on, reviewed . . . 46 *et seq.*
, distinction between death and . . . 48
, phenomena of death from . . . 50
Atomic weights, Dr. Turner's experiments on . . . 489
Autopsy by steam . . . . . . 230

Balfour (Dr. Wm.) on Manual Operations in the Cure of Rheumatism, &c. reviewed . . . . . 100 *et seq.*
Bar at the neck of the bladder . . . . 31
Baudelocque (A. C.) on Internal Uterine Hemorrhage, reviewed 285 *et seq.*
Beck (Dr. E.) on Lepra and Psoriasis, reviewed . 371 *et seq.*
Belladonna in Hooping-cough . . . . 416, 464
Bellingeri's account of the Functions of the Nerves . . 424
Bichat's theory of Asphyxia erroneous . . . 53
Bladder, Mr. Guthrie on the Neck of the . . . . 7
, Mr. Guthrie's method of puncturing the . . 25
Bleeding, best method of . . . . . . 276
Blood, scarlet, may issue from a vein . . . 284
Boivin and Dugès on Diseases of the Uterus, translated by Mr. Heming, reviewed . . . . . . 33 *et seq.*
Bones, Dr. Witt's mode of preparing, for osteological purposes . 386
Bourgery on the Minor Surgical Operations, reviewed . 275 *et seq.*
Bow (Dr.) on Inflammation, reviewed . . 109 *et seq.*

Brain; its natural appearance . . . . . 59
Dr. Abercrombie on Diseases of the, reviewed . 301 *et seq.*
Burial in honey . . . . . . 238

Calomel and cold water in Cholera . . . . 147
Cantharides; their effects when internally administered . 151
Capivi in Catarrh of the Bladder and Leucorrhœa . . 475
Carbutt's Clinical Lectures, reviewed . . . 308 *et seq.*
Carditis, Dr. Stroud's cases of . . . 187, 439
Carotid Artery and Internal Jugular Vein tied . . 219
Carswell's Illustrations of the Elementary Forms of Disease (Fasciculus VI. Hemorrhage,) reviewed . . . 298 *et seq.*
Cataract, method of making the incision in extraction of the . 205
Catheter, Dr. Maunsell's method of passing the female . . 114
Cavity on the surface of the Brain, case of a . . 307
Cerebellum, case of tubercular disease of the . . . 303
Charitable establishments at Florence . . . 486
Chitty on Medical Jurisprudence, reviewed . . 104 *et seq.*
Cholera not yet tolerably treated . . . . 413
Chrisome; an entry in the old Bills of Mortality . . 493
Circulation, Dr. W. Philip on the Sources and Nature of the Powers of the 85
Clinical Instruction, Louis on, reviewed . . . 408
Lectures, by Dr. Carbutt, reviewed . . 308 *et seq.*
Club-foot cured by division of the Tendo Achillis . . 214
College of Physicians, income of the . . . . 239
Colours of Plants, cause of the . . . . 488
Conjunctiva, scrofulous inflammation of the . . . 330
Cotton-mills . . . . . . 231
*Corde*; meanings of this word . . . . 159
Corrosive Sublimate, case of poisoning by . . 69
Creosote, account of . . . . . 417
Crotchet, use of the, unadvisable . . . 117
Croup, cases of, cured by an opiated liniment . . . 109
Cutler's Surgeon's Practical Guide in Dressing, &c., reviewed 405 *et seq.*

Dalrymple (John, Esq.); his account of two English mummies . 169
Davies (Dr. Henry); Cases extracted from the Note-book of . 166, 430
Death, Dr. W. Philip on the Nature of . . . 66
Delirium Tremens, Dr. Roberts' case of . . . 198
Demonstration of the Nerves, by Swan, (4to. Edition,) reviewed 140 *et seq.*
Diabetes, cases of, treated by Dr. Peacock . . 144
insipidus, cases of . . . . . . 312
mellitus, cases of . . . . . 313
Diagnosis, difficulty of . . . . . . 482
Diarrhœa in infants, treatment of . . . 218
Dictionary of French Medical Terms, by Dr. Shirley Palmer, reviewed, 156 *et seq.*
Digestion, Dr. W. Philip's observations relating to the Function of 81
Dispensary Abuses, reviewed . . . . . 162
Druggery, Mr. Everest's observations on . . . 401
Dublin Practice of Midwifery, by Dr. Maunsell, reviewed . 113 *et seq.*
Dupuytren on Wounds by Military Weapons, vol. I., reviewed 120 *et seq.*
vol. II., reviewed 339 *et seq.*

Eden's Sciential Medicine, reviewed . . . . 410
Emetics often useful in Dyspepsia . . . . 311
Entozoa found in the eye, case of . . . 336
Entries in the old Bills of Mortality . . . . 493
Ergot of Rye, Dr. Maunsell on the . — . . 116
used in a case of slow labour . . . 432
retained placenta . . 463

Everest's Popular View of Homœopathy, reviewed . 29 *et seq.*
Excretions of plants . . . . 492
Extirpation of a Tumour of the Neck . . 219
Eye, insensibility of the . . . . 204
its sensibility to light . . . . . 319
Mackenzie on Diseases of the, reviewed . . 324 *et seq.*

Falsification of writings . . . . . . 494
Fellowship of the College of Physicians, disqualifications for the . 240
Fifth pair of nerves, third trunk of the, described by Mr. Swan . 141
Fistulous opening of the stomach, case of . . . 484
Fœtus in utero, crying of the . . . . . 217
, physiology of the . . . . . 226
Foote's Medical Pocket-book for 1835, reviewed . . 407
Fractions, books should not be published in infinitesimal . 227
Fracture, case of ununited, successfully treated by friction . . 467
Fragmenta de Viribus Medicamentorum Positivis, a Samuele Hahnemann, M.D., reviewed . . . . . 149 *et seq.*
Fungus hæmatodes, case of, successfully treated . . . 464

Galvanism and acupuncture . . . . . 472
Gastrodynia sometimes benefited by carbonate of iron and compound powder of jalap . . . . . . . 273
often benefited by large doses of alum . . ib.
Gastro-enteritis often accompanies phthisis . . . 308
Generation; its varieties . . . . . . 362
Ginseng . . . . . . 228
Good's (Mason) Study of Medicine (fourth Edition,) reviewed 391 *et seq.*
Graefe's apparatus for passing a ligature round polypi . . 422
Granville's (Dr. A. B.); his account of the physiology of the fœtus . 226
his method of treating relaxed uvula . 392
Griffin on Functional Affections of the Spinal Cord, reviewed 243 *et seq.*
Grinders' magnetic guards but little used . . . 379
Gunshot Wounds of the Abdumen, Dupuytren on . . 249
Guthrie on Diseases of the Bladder, reviewed . . 3 *et seq.*

Hæmoptysis, case of apparent, caused by a leech . . 460
Hahnemanni Fragmenta de Viribus Medicamentorum Positivis, reviewed, 149 *et seq.*
Halford (Sir H.) on the Studies of a Physician . . . 232
Hannibal, the death of . . . . . 495
Hastings' Illustrations of the Natural History of Worcestershire, reviewed, 376 *et seq.*
Headach, pills for . . . . . . 149
Heart wounded by a hair-pin . . . . 124
, case of malposition of the . . . . 213
Hemorrhage, Dr. Carswell on . . . . 298 *et seq.*
Cerebral, case of . . . . 299
Urethral, may give rise to retention of urine . 300
in the Digestive Organs; its characters . . ib.
Dupuytren on . . . . 339
from the Cæcum, case of, by Mr. F. E. Hicks . 457
Hepatic abscess, case of . . . . 483
Hicks' (Mr. F. E.) case of Hemorrhage from the Cæcum . 457
Himalayan plants, Royle's, reviewed . . 388 *et seq.*
Hippocrates; the results of his practice misunderstood, by Sir G. Blane 149
Homœopathy, Everest's Popular View of, reviewed . . 399 *et seq.*
the Editor's observations on . . . 414
Hope's Morbid Anatomy, reviewed . . . 58 *et seq.*
Horse-chesnut; characters of its fecula under the microscope . 139
Hospital gangrene . . . . . 345

Howship (Mr. J.) on two cases of Inflammatory Tumour, produced by the Larva of the Œstrus Humanus . . . . . 174
Hunter (John); his view of living matter . . . . 359
Hydatids, case of . . . . . . 148
of the kidneys passed by the urethra . . 224
Hydrated oxide of iron, supposed to be an antidote to arsenic . 417
Hypochondriac, a sturdy . . . . 227
Hysteria imitates almost every disease . . . 244
Hysterical knee, cases of . . . . . 461

Illustrations of the Effects of Poisons, by Dr. Roupell, reviewed 67 *et seq.*
Illustrations of the Elementary Forms of Disease, by Robert Carswell, M.D. reviewed . . . . . . . 298 *et seq.*
Inflammation, Dr. Bow on, reviewed . . . 109 *et seq.*
Inquiry into the Nature of Sleep and Death, by Dr. A. P. W. Philip, reviewed . . . . . . . 70 *et seq.*
Iodine exists in Cheltenham water . . . . . 311
in Syphilis . . . . . . 416
Ioduret and Hydriodate of Iron, Dr. A. T. Thomson on the, reviewed, 60 *et seq.*
objection to their use . . 419
Iris; is its structure muscular? . . . . . 321
Itch insect discovered . . . . . 417, 466

Jamaica Physical Journal, edited by James Paul, Esq., reviewed 159 *et seq.*
Jobson on Caries of the Teeth . . . . . 202

Kay (Dr.) on Asphyxia, reviewed . . . 46 *et seq.*
Kiernan's account of the Anatomy of the Liver . . 396
Kilgour's Lectures on the ordinary Agents of Life, reviewed 402 *et seq.*
King's (Mr. Thomas) Remarks on Aneurisms of the Cerebral Arteries; with Cases . . . . . . 434

Laudations of new remedies must be listened to with caution . 147
Lectures on the ordinary Agents of Life, by A. Kilgour, M.D. reviewed, 402 *et seq.*
Leeches, the application of . . . . . 281
Lepra and Psoriasis, Dr. Beck on, reviewed . . 371 *et seq.*
, cases of, cured by tar pills . . . 372 *et seq.*
Leroy's bellows recommended for insufflation, by Dr. Kay . . 49
method of galvanising asphyxiated subjects . . 56
Lithotrity, the Editor's observations on . . . 421
is as fatal as Lithotomy . . . 422
Longevity, instances of, in Worcestershire . . . 382
Louis on Clinical Instruction, reviewed . . . 408
Lupus, treatment of . . . . . 149

Mackenzie on Diseases of the Eye, reviewed . 324 *et seq.*
Mandrake . . . . . 238
Marasmus, case of . . . . . . 166
Maunsell's Dublin Practice of Midwifery, reviewed . 113 *et seq.*
Mead quoted . . . . . . 227, 8
Medical Jurisprudence, Chitty's Practical Treatise on, reviewed 104 *et seq.*
education, defects in . . . . 236
Almanack for 1835, reviewed . . . . 407
Pocket-book for 1835, by Mr. Foote, reviewed . . ib.
responsibility in France . . . 237
and Chirurgical Society; its charter . . . 401
Meningitis, chronic, remarkable case of . . . 301
Merriman, Dr. S.; his observations on the treatment of Diarrhœa in Infants, 218
Meteorological Register . . . . . 242, 498
Microscope; Raspail's great instrument of discovery . . 136
Middle meningeal artery, case of rupture of the . . 306

Mineral waters, Dr. Daubeny's observations on . . . . 312
Moon, influence of the . . . . . 228
Mortification, case of . . . . . 143
Mummies . . . . . . 229
, manufacture of . . . . . 230
, account of two English, by Mr. Dalrymple . 169
Muriate of antimony; its effects in carcinoma . . . 469
Muscles about the bladder; new ones discovered by Mr. Guthrie . 14
, case of choleroid affection produced by . . 179

Neck of the femur; diagnosis of its fractures . . 477
Nerves of the lungs, effects of dividing the . . . 78
, Bellingeri's account of the functions of the . 424
Nervous system; Dr. A. P. W. Philip on its functions . . 71
and muscular systems, on the relation which subsists between the 89
Notices . . . . . . 242, 498
Numerical method, M. Louis on the advantages of the . 408

Oculists in the seventeenth century . . . . 233
Œstrus Humanus; account of two cases of inflammatory tumour produced by its larva . . . . . 174
Ophthalmia, purulent, Mr. Walker's treatment of . . 314
Dr. Mackenzie's account of . . . 327
subdivision of . . . 335
neonatorum . . . . 332
, prognosis of . . . 333
phlyctenular, case of . . . 331
Ophthalmic Surgery. John Walker on the Principles of, reviewed 314 *et seq.*
Opium; its use in mania . . . . 485
Organic Chemistry, New System of, by Raspail, translated by Henderson, reviewed . . . . . 135 *et seq.*
Ossification of the muscular tissue, case of . . . 222
Osteology, Witt's Compendium of, reviewed . . 348 *et seq.*
Oxalic acid, case of poisoning by . . . . 68

Pain from exhaustion . . . . . . 145
Palmer's (Dr. S.) Dictionary of French Medical Terms, reviewed 156 *et seq.*
Paralysis; its relation to the seat of effusion in the brain . . 298
Peacock's Practical Hints, reviewed . . . 143 *et seq.*
Pericranium, on certain affections of the . . . 305
injury of the, cured by incision . . ib
Perineum, suture of the . . . . . 205
Philip (A. P. W.) on the Nature of Sleep and Death, reviewed 70 *et seq.*
Phlegmasia Dolens, Dr. Maunsell's observations on . . 119
Phosphorescence of vegetables . . . . 370
Phthisis, mortality from, at different ages . . . 492
often complicated with ulceration of the bowels . 309
Physical effects of projectiles from firearms, Dupuytren on the . 133
Physician, the studies of a . . . . . . 232
Physicians, classic; their opinions misrepresented in modern books 399
Physiology, Tiedemann's Comparative, reviewed . 351 *et seq.*
Piles, divided, by Dr. Carswell, into two kinds . . 301
Placenta, cases of retained, by Dr. H. Davies . . . 430
adhering to the fundus of the uterus . . 463
Platysma myoides, Sir C. Bell on the . . . 491
Polypi, treatment of nasal . . . . 465
Porrigo favosa, treatment of . . . . 395
Portio dura, cases of paralysis of the . . . 216
Practical Hints, by Dr. Peacock, reviewed . . 143 *et seq.*
Principles and Illustrations of Morbid Anatomy, by Dr. Hope, reviewed, 58 *et seq.*
Prostate, female, discovered by Mr. Guthrie . . . 13
gland, Mr. Guthrie on the . . . 9

Prussic acid, Laming's method of preparing . . . 421
Psoriasis, Dr. Beck on . . . . . 375
Pulse in horses . . . . . . . 234
Punctured wounds through soft parts, Dupuytren on . 121
Punctures through the harder substances, Dupuytren on . . 122
Pupil, artificial, Mr. Tyrrell on the formation of, . . . 424

Quain's Anatomical Plates . . . . . 227
Elements of Anatomy reviewed . . 396 *et seq.*

Rabies in the horse, case of . . . . 237
Raspail's Organic Chemistry, translated by Henderson, reviewed 135 *et seq.*
Rat, old English black, still found in Worcestershire . 383
Respiration, relation of the sensorial power to, . . 76
Retinitis . . . . . . 323
Retrospect of the late Improvements in Medicine, surgery, &c. by the Editor 413
Roberts' (Dr. C. J.) case of Delirium Tremens . . 198
Roupell's Illustrations of the Effects of Poisons reviewed . 67 *et seq.*
Royle's Himalayan plants (Part IV.) reviewed . 388 *et seq.*
Respiration of vegetables . . . . . 365

Sarracennia . . . . . 236
Scarlatina, case of, followed by serous effusion . . 303
Sciential Medicine, Mr. Eden on, reviewed . . 410
Scott on Tic douloureux, reviewed . . . 152 *et seq.*
Scrofula, case of . . . . . . 168
Senses can be educated . . . . . . 409
Shepherd's forceps for extracting stumps of teeth . 422
Short-sightedness, Mr. Walker's opinions on . . . 320
Shoulder, dislocations of the, method of reducing by the heel in the axilla 211
Simpson's Anatomy for Artists, reviewed . . . 161
Sleep, Dr. W. Philip on the Nature of . . . . 92
Smilax aspera, a cheap substitute for sarsaparilla . . 420
Soemmering's foramen . . . . . 326
*Speculum uteri*; its disadvantages . . . . . 36
Spine, Griffin on Functional Affections of the, reviewed 243 *et seq.*
cases of functional diseases of the . 246, 250, 252, &c.
table of 148 cases of functional diseases of the . . 271
method of treating functional diseases of the . . 273
functional diseases of the . . . . 415
Squinting, case of, . . . . . . 392
Stanley's (Dr.) ingenious theory of Animal Magnetism . 459
Starch; its characters under the microscope . . 137
Stethoscope, the, must not supersede the ordinary modes of examination 35
Strangulated hernia, case of, followed by cholera . . 460
Strata, effects of, upon health . . . . . 379
Stricture of the Urethra; its usual seat . . . . 17
Stricture, effects of over-distending a . . . . 22
best method of dilating a . . . . . 28
Stroud's (Dr. Wm.) cases of Carditis . . . 187, 439
Study of Medicine, Mason Good's, reviewed . . 391 *et seq.*
Sulphuric acid, case of poisoning by . . . . 67
Surgeon's Practical Guide in Dressing, &c., Dr. Cutler's, reviewed 405 *et seq.*
Surgical operations, Bourgery's treatise on the minor, reviewed 275 *et seq.*
Swan's Demonstration of the Nerves, (4to. Edit.) reviewed 140 *et seq.*
Syphilis treated with iodine . . . . . 416

Taliacotian operation, case of, by Mr. Tyrrell . . 448
Tannin, a combination of resin and gallic acid . . 140
Tar-pills recommended in lepra, by Dr. Beck . . 371
Tar-water, extravagant panegyrics lavished on, by Bishop Berkeley . 418

Tea plant; its varieties . . . . . 388
Technical terms, French, where explained . . . . 157
Teeth, caries of the . . . . . . . 202
Temperament . . . . . . . . 235
Tendo Achillis divided, for the cure of club-foot . . 214
Tetanus, aphorism of Hippocrates on . . . . . 125
Thompson's (Dr. Theophilus) account of a case of Choleroid Affection produced by the Poison of Muscles . . . . 179
Thomson (A. T.) on the Ioduret and Hydriodate of Iron, reviewed 60 *et seq.*
Thymus Gland, Dr. Bow on the use of the . . 112
Sir A. Cooper's account of its anatomy and physiology 422
Tic Douloureux, Scott on, reviewed . . 152 *et seq.*
cases of, . . . . . 153 *et seq.*
treated with ointment of ioduret of mercury . 155
Tiedemann's Comparative Physiology, reviewed . . 351 *et seq.*
Torsion of Arteries, Dupuytren's observations on the, . 342
Trachea and Œsophagus, case of a wound of the . . 209
Training, Dr. Kilgour's account of, . . . 404
Trepanning, Dupuytren on . . . . . 347
Treviranus; his view of living matter . . . 359
Turning, method of, in placental presentations . . 118
Typhus fever, case of, suddenly cured by fright . . 271
Tyrrell (Mr. F.) on the Formation of Artificial Pupil . . 424
on the Taliacotian operation . . . 448
Tænia, case of, discharged from the meatus urinarius . . 392

Ureters, Mr. Guthrie's account of the . . . 4
Urethra, length of the . . . . . . 16
cases of disease of the . . . . . 19
hemorrhage from the . . . . . 23
Uterine Hemorrhage, internal, Baudelocque on, reviewed 285 *et seq.*
its various seats and periods . 285, 288
cases of . 285, 286, 287, *et seq.*
symptoms of . . . 289
Uterus, super-pubic examination of the . . . 38
examination per vaginam of the . . . 38
examination per rectum of the . . . 39
anteversion of the . . . . . 39
cancer of the . . . . . 42
excision of the . . . . . . 45
its muscular fibres . . . . . 115
Perineum, and Rectum, case of their destruction after delivery 480
Uvula, relaxed, method of treating . . . 392

Vaccination, accidental . . . . . . 494
Vale of the Severn, mildness of the temperature of the, . 384
Vegetation near Quito . . . . . 490
Veins, Mr. Chitty's account of the . . . 107
Veins, cases of the admission of air into the . . . 206
Veratria, the Editor's observations on . . . . 420
Vindication of the pre-eminent Efficacy of Manual Operations in the Cure of Rheumatic and Nervous Diseases, by Wm. Balfour, M.D., reviewed, 100 *et seq.*
Visceral abscesses, or deposits of pus . . . . 343
Vital Heat, Tiedemann on . . . . . . 368

Walker's (J.) Principles of Ophthalmic Surgery, reviewed . 314 *et seq.*
Wheat-flour at Paris is commonly adulterated . . . 139
Witt's Compendium of Osteology, reviewed . . 384 *et seq.*
Wolff's revolving bedstead . . . . . . 422

Worcestershire, Dr. Hastings' Illustrations of the Natural History of, reviewed . . . . . . 376 *et seq.*
Wounds, Dupuytren on the healing of, by the first intention . 126
dressing of . . . 127
union of, after suppuration . . 128
lacerated . . . . 130
of the head, Dupuytren on . . . 346
of the face, case of . . . . 348
case of recovery after severe . . . 160
Young (Dr. Thomas) observed that phthisis is often complicated with ulceration of the bowels . . . . . . 309

---

### PLATE.

Two English Mummies, found at Wymondham, near Norwich, *to face page* 169

